# Long non-coding RNA CATIP antisense RNA 1 (lncRNA CATIP-AS1) downregulation contributes to the progression and metastasis of thyroid cancer via epithelial–mesenchymal transition (EMT) pathway

**DOI:** 10.1080/21655979.2022.2047400

**Published:** 2022-03-10

**Authors:** Fujian Qi, Ji’Ao Tang, Zhenling Cai, Gang Wang, Zhijun Wang

**Affiliations:** Department of General Surgery, The Fifth Affiliated Hospital of Southern Medical University, Guangzhou, Guangdong Province, China

**Keywords:** CATIP-AS1, thyroid cancer, EMT, miR-515-5p, smad4

## Abstract

Thyroid cancer (THCA) is the most common cancer of the endocrine system across the globe. To date, the mechanism of development of THCA remains scarcely known. In this study, we aim to elucidate the long non-coding RNA CATIP antisense RNA 1 (lncRNA CATIP-AS1/CATIP-AS1) role in the pathogenesis of THCA and its regulatory mechanism. The result shows that the CATIP-AS1 was significantly downregulated in THCA tissues and cells and was associated with a poor prognosis of patients diagnosed with THCA. The overexpression of CATIP-AS1 significantly inhibited THCA cell proliferation, migration, and epithelial–mesenchymal transition (EMT) but increased the THCA cell apoptosis. We found that CATIP-AS1 endogenously sponges miR-515-5p and its overexpression could inhibit miR-515-5p regulatory effect. Moreover, the overexpression of miR-515-5p repressed the Smad4 expression level, consequently reversed the inhibiting effect of overexpressed CATIP-AS1 on the proliferation, and migration of THCA cell. It also reversed the increased THCA cell apoptosis and the downregulated-CATIP-AS1-induced cell EMT inhibition. Summarily, we demonstrated that the CATIP-AS1 promotes the progression and metastasis of THCA via EMT pathway partly through regulating the miR-515-5p and Smad4 expression in THCA cell. The CATIP-AS1 could be a promising biomarker for early THCA detection and prognosis and a possible therapeutic target for its treatment.

## Graphical Abstract

**Figure:**
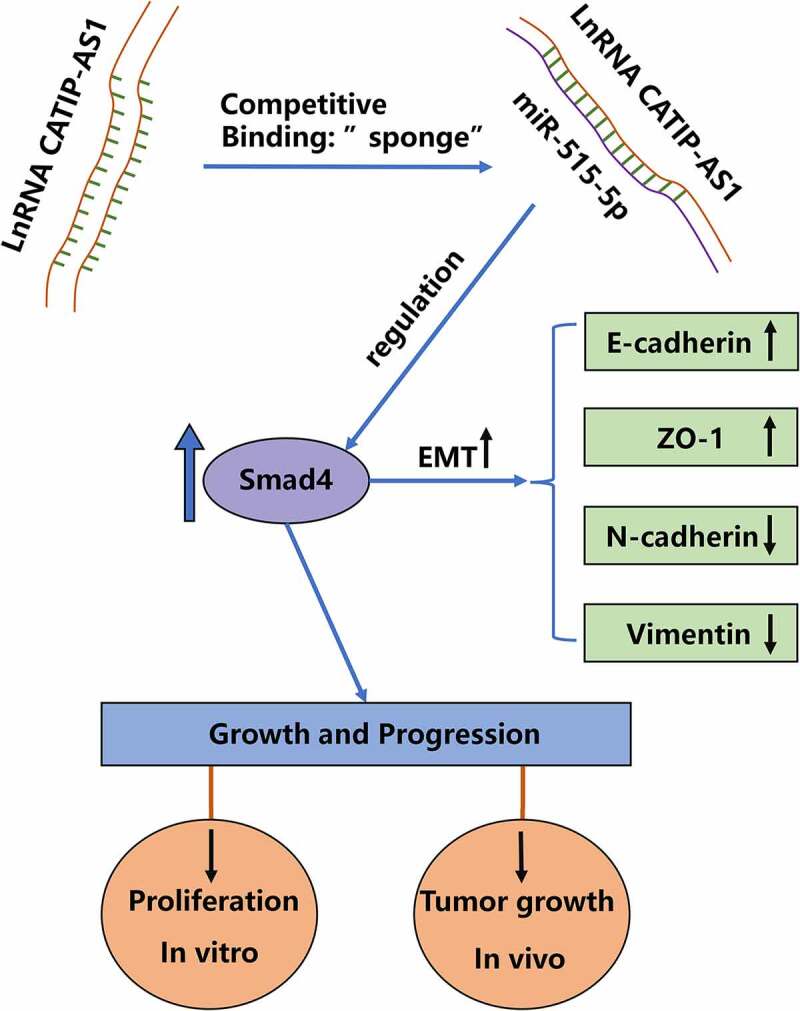

## Introduction

THCA is a pervasive cancer of the endocrine system, with a rapid worldwide increase in the number of recorded cases over recent decades. About 185% increase in the incidence rate of THCA was observed globally between 1999 and 2017, with China, the US, and India having the highest number of cases, respectively [1]. To date, no effective treatment has been established for THCA patients with either a poorly differentiated papillary thyroid cancer (PTC) or anaplastic thyroid cancer (ATC), which is a rare, but very aggressive, human malignant tumor [2]. Hence, there is an urgent need to explore a new molecular target for THCA treatment. Moreover, the treatment of THCA has been limited to total and partial thyroidectomy, which may also include the removal of the surrounding nymph node that might likely affect the vocal cords of THCA patients. Besides, early diagnosis of THCA cases still remains quite hard to get and the identification of molecular biomarkers for early diagnosis and prognosis of THCA is very crucial to patient’ survival. Regulatory molecules like the non-coding RNAs have, however, since been shown as potential biomarkers for THCA [3–5].

LncRNAs are RNA molecules that lack the ability to be translated into proteins. Accumulating evidence shows that lncRNAs are key regulatory molecules involved in the development and pathogenesis of various human diseases as competing endogenous RNAs (ceRNAs), including cancer [6–9], diabetic retinopathy [10,11], coronary atherosclerotic heart disease (CAD) [12,13], and pre-eclampsia [14,15] through lncRNA-miRNA-mRNA network. For instance, LINC01134 accelerates hepatocellular carcinoma progression by sponging miRNA-4784 and downregulating structure-specific recognition protein 1 [16]. Interestingly, this mechanism also plays an important role in the progression of THCA. For example, lncRNA XIST promotes migration and invasion of PTC cell by modulating miR-101-3p/CLDN1 axis [17]. LncRNA-HCG18 regulates the viability, apoptosis, migration, invasion, and epithelial–mesenchymal transition of PTC cells via regulating the miR-106a-5p/PPP2R2A axis [18]. Similarly, lncRNA MCM3AP-AS1 promotes proliferation and invasion through regulating miR-211-5p/SPARC axis in PTC [19]. The chromosomic location of CATIP-AS1 is chr2:218,366,665–218,367,835, and this gene is less reported in cancer. Interestingly, Jianhong Wang et al. revealed that the expression of CATIP-AS1 in THCA is significantly down-regulated [20]. This is a novel finding that lays the foundation for our research. However, in-depth study of the signal pathway of CATIP-AS1 in regulating THCA was still lack; therefore, the specific molecular mechanism of CATIP-AS1 in THCA needs to be explored urgently.

Therefore, the purpose of the present study was to explore CATIP-AS1 role in the progression of THCA and its potential as a new molecular target for the treatment of THCA. These findings will provide the basis for the molecular mechanism of CATIP-AS1 on the effect of THCA, which supports CATIP-AS1 might act as an important gene in the treatment of THCA. As a result, we found that CATIP-AS1 is significantly down-regulated in THCA tissues and related to poor prognosis. For the first time, we reported that the CATIP-AS1 is a tumor suppressor that regulates the progression and metastasis of THCA cell via the EMT pathway.

## Materials and methods

### Analysis of thyroid cancer tissue datasets and clinical samples

Normal THCA tissues and adjacent healthy ones were purchased from 60 individuals each after surgical resection. The samples were gathered from patients admitted at The Fifth Affiliated Hospital of Southern Medical University following the endorsement of the Hospital’s Ethics Committee (approval number: [IR-B-2020-11-21]) from November 2018 to September 2020. Immediately after the clinical tissue samples were collected, they were snap-frozen and kept at −80°C for subsequent experimental and analytical work [21]. Furthermore, GEPIA analysis of CATIP-AS1 expression was performed in THCA tissue sample data.

### Cell culture

Human normal thyroid (Nthy-ori3-1) cell lines and THCA cell lines (BCPAP, IHH4, 8505C, and BHT-10) were obtained from the American Type Culture Collection (ATCC) and then cultured in the medium-RPMI-1640 (Hyclone) together with 10% and 1% of FBS and penicillin-streptomycin (Sigma), respectively. Cells were preserved in 5% CO_2_ humidified incubator at 37°C [22].

### Transfection of thyroid cancer cell line

pcDNA3.1-CATIP-AS1 overexpressing plasmid and empty vector were synthesized commercially by GenePharma (Shanghai, China) following the producer’s recommended protocol. Concisely, THCA cell lines were seeded into a12-well plate (1 × 10^5^ cells/well) and later transfected with various oligonucleotides purchased from Genepharma (Shanghai, China), including miRNA mimics (5’-GUCUUUCACGAAAGAAAACCUCUU-3’) and its negative control (miR-mimics-NC: 5’-CAGUACUUUUGUGUAGUACAA-3’;); inhibitors (5’-CAGAAAGUGCUUUCUUUUGGAGAA-3’) and negative control (miR-inhibitor-NC: 5’-CGAACGUGUCACGUTT-3’). Lipofectamine™ 3000 Transfection Reagent (Takara, Kusatsu, Japan) was performed to transfect the plasmids [23].

### RNA extraction and quantitative real-time polymerase reaction (qRT-PCR) analysis

TRIzol reagent (Invitrogen) was used for total RNA extraction from clinical samples and cells following the manufacturer’s guide. After, the RNA was quantified using a One‑Step SYBR Prime Script RT‑PCR kit (Takara) [21]. The miR-515-5p, CATIP-AS1, Smad4 expression level was calculated using the 2^−ΔΔCt^ method and the GAPDH and U6 were used as the endogenous controls [17]. The experiment was done in triplicate.

### CCK-8 and colony formation assay

Cell proliferation and viability analysis was done with CCK-8 kit (Beyotime). Briefly, after transfection, the BCPAP and BHT-101 cells were put into 96-well plates (5 × 10^3^ cells/well), CCK-8 solution (20 μL) was added and then incubated for 2 hours. On a microplate reader, the absorbance was calculated at a wavelength of 450 nm [24]. In the colony formation assay, BCPAP and BHT-101 cells were trypsinized, seeded in plates, and then incubated for 5 days at 37°C. The colonies were dyed using a dyeing solution comprising 20% methanol and 0.1% crystal violet (Beyotime, Biotechnology) [25]. After which, the colonies were counted and tested.

### Apoptosis assay

Into a previous culture collection medium (Beyotime), cells were re-suspended and later transferred into a fresh centrifuge tube. After, the cells were re-suspended gently in pre-cooled 1X binding buffer (0.5 mL), then incubated with Annexin-V FITC (5 μL) and PI (10 μL) (Beyotime) for 15 minutes in the dark. Cell evaluation was done with flow cytometry (Beckon) [26].

### Transwell assay

The cells were seeded on the polycarbonate Transwell filters (Sigma) coated with Matrigel for invasion analysis. The cell suspension was done in a medium with and without serum and then incubated for 48 hours at 37°C. The noninvasive cells were removed with cotton swabs, and the invaded cells were fixed in methanol (100%) for 10 minutes, then air-dried, and stained with crystal violet solution [27]. The counting was done under a microscope.

### Western blot analysis

Extraction of the total protein was finished with RIPA kit (R0010, China). The protein was separated with SDS-PAGE (10%), moved to PVDF membrane, and later blocked with Tris-buffered saline with Tween 20 solution comprising 5% BSA (Thermo fisher). Then, the membrane was incubated with primary antibodies to E-cadherin (cat. 3195S, Cell Signaling Technology/CST), ZO-1 (cat. 13663S, CST), N-cadherin (cat. 5741S, CST), vimentin (cat. 13663S, CST) and CATIP-AS1 at 4°C overnight. After that, the membrane was incubated with a secondary antibody to HRP-labeled Goat Anti-Rabbit IgG (H + L) (cat. A0208, Beyotime, Beijing). Blots were viewed with electrochemiluminescence (ECL) chromogenic substrate [28].

### Xenograft experiments

BCPAP cells (3 × 10^6^) transfected with pcDNA3.1-CATIP-AS1 vector and control empty vector were subcutaneously injected into the flank of 6-week BALB/c nude mice. The animal experiment was approved by the ethics committee of the Fifth Affiliated Hospital of Southern Medical University. Tumor volume was measured every 7 days and calculated according to the formula: Tumor volume (mm^3^) = (width) × (height)2/2. After 28 days, the mice were sacrificed and tumor weights were measured.

### Bioinformatic prediction of target gene

The expression profiles of CATIP-AS1 in THCA tissues from The Cancer Genome Atlas (TCGA) were analyzed using GEPIA. After, Lncbase and Starbase tools were used to predict CATIP-AS1 target miRNA and miR-515-5p target mRNA [29].

### Luciferase reporter assay

Mutated and wild-type CATIP-AS1 and Smad4 sequences were cloned into the luciferase reporter vector (Promega). BCPAP and BHT-101 cells were co-transfected with the luciferase reporter vectors comprising CATIP-AS1 and Smad4 sequences, and miR-515-5p and miR-negative controls. After 48 hours, the relative luciferase activity was measured with a luciferase assay kit (Promega) in accordance with the manufacturer’s guide. Renilla luciferase activity was invoked as the normal control [30].

### Biotinylated RNA pull-down assay

The THCA cell lines (BCPAP and BHT-101) transfected with biotinylated oligonucleotides were lysed after 48 hours with specific cell lysis buffer (Ambion). This is followed by the incubation of the cell lysate with M-280 streptavidin magnetic beads (Sigma) pre-coated with yeast tRNA and RNase-free (Sigma) for 3 hours at 4°C. Then, cold lysis buffer, low- and high-salt buffer was utilized to wash the cells. After, TRIzol reagent was used for total RNA extraction, while the expression of miR-515-5p was detected through qRT-PCR analysis [31].

### RNA immunoprecipitation (RIP)-qRT-PCR

CATIP-AS1 binding to Argonaute-2 protein (Ago2) was marked with RIP kit (Millipore). RIPA lysis buffer (Beyotime) was used for cell lysis. A fraction of the cell lysate was stored as an input, while the remaining fraction was co-precipitated with antibody. After washing, re-suspension of the magnetic-antibody complex in RIP wash buffer (900 μL) was done and then incubated with cell lysate (100 μL) overnight at 4°C. After, the samples were placed on a magnetic base for magnetic base–protein complex collection. Extracted RNA from the precipitated sample was interpreted with proteinase K for PCR analysis. Polyclonal antibody against Ago-2 was utilized for RIPA together with anti-human antibody against IgG. After, the enrichment of miR-515-5p in the CATIP-AS1 probe was evaluated via qRT-PCR [30].

### Statistical analysis

Data for the experiments were shown as the mean ± SD. The statistical analyses were finished with SPSS 22.0 (Chicago). Also, differences between the two experimental groups were analyzed using student’s t-test. While significant statistical differences were observed at p < 0.05 [32].

## Results

In our study, we hypothesized that CATIP-AS1 downregulation might contribute to the progression and metastasis of THCA via EMT pathway. To verify this hypothesis, we first verified the expression level of CATIP-AS1 in THCA. Second, we conducted the CATIP-AS1-overexpression model in BCPAP and BHT-101 cell lines to investigate the function of CATIP-AS1 in THCA cell proliferation, apoptosis, and metastasis and reveal the intrinsic molecular mechanisms. Furthermore, miR-515-5p was predicted to be the target gene of CATIP-AS1, and SMAD4 was the target mRNA. Ultimately, we detected the expression of EMT-related protein to confirm the EMT pathway regulated by CATIP-AS1 on the miR-515-5p/SMAD4 axis.

### The CATIP-AS1 is downregulated in thyroid cancer samples and associated with poor prognosis of patients

**Figure 1.**
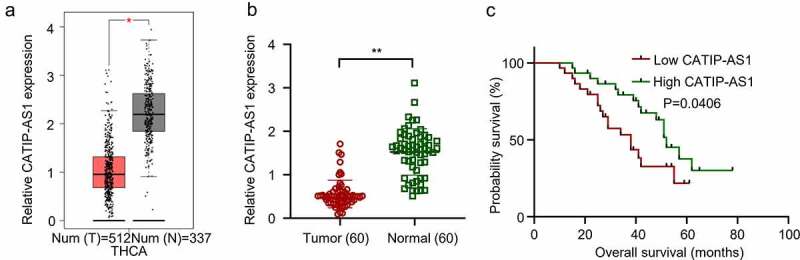
The differential expression analysis of CATIP-AS1 in tissue samples (a) GEPIA analysis of CATIP-AS1 expression in THCA tissue samples. CATIP-AS1 was significantly downregulated in thyroid samples relative to the normal ones. (b) qRT-PCR analysis of CATIP-AS1 expression in clinical tissue samples. CATIP-AS1 expression was significantly downregulated in THCA tissue samples compared to the adjacent normal ones. (c) Statistical analysis of the relationship between CATIP-AS1 expression level and THCA patients’ survival time. Low expression of CATIP-AS1 was associated with THCA patients’ poor prognosis.

A total of 512 publicly available THCA datasets were analyzed using GEPIA. The results indicated that the CATIP-AS1 is significantly downregulated in THCA tissues compared to the normal healthy ones (*p* < 0.05, Figure 1(a)). Utilizing 60 THCA clinical samples and adjacent normal thyroid tissues, we determined CATIP-AS1 aberrant expression through qRT-PCR analysis. As shown in Figure 1(b), CATIP-AS1 expression was significantly downregulated in THCA tissues samples relative to the adjacent matched ones (*p* < 0.01). This result is also consistent with the one obtained from the GEPIA database analysis. Interestingly, we found that CATIP-AS1 low expression level was associated with poor survival rate of THCA patients (*p* = 0.0406, Figure 1(c)). Collectively, this data indicates that the low expression level of CATIP-AS1 in THCA tissue is associated with poor prognosis of THCA patients.

### *CATIP-AS1 downregulation promotes* in vivo *tumor progression in thyroid cancer cell lines.*

**Figure 2.**
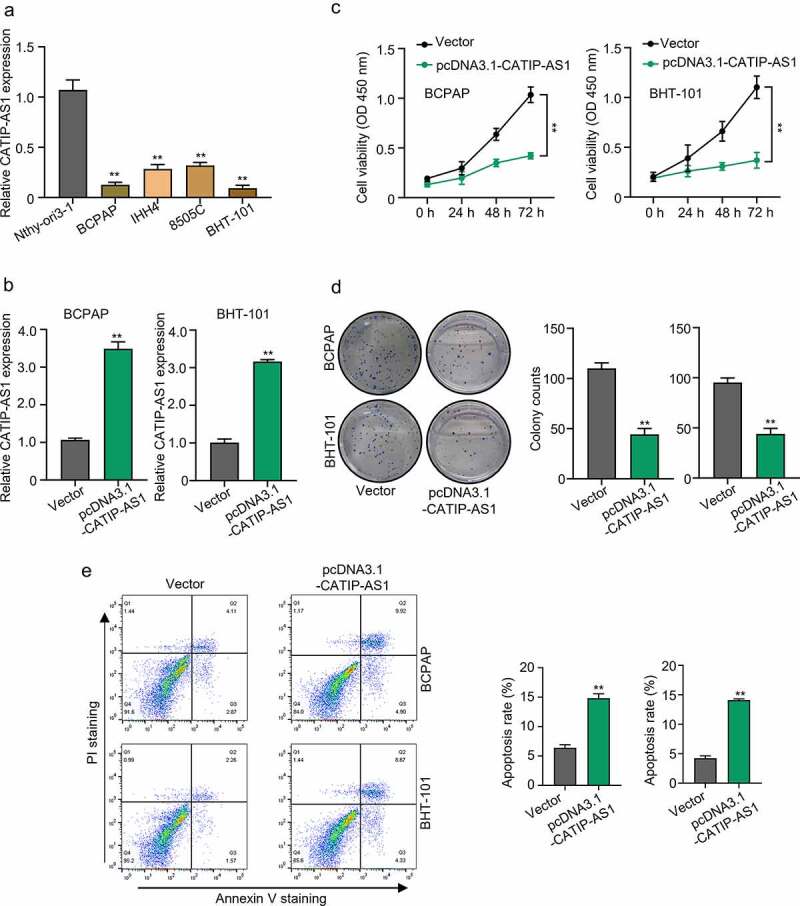
Functional study of CATIP-AS1 in THCA cells (a) The relative expression of CATIP-AS1 in different THCA cell lines and normal human thyroid cell line, Nthy-ori3-1, determined by qRT-PCR analysis (b) CCK-8 analysis of THCA cell proliferation after overexpressing CATIP-AS1. CATIP-AS1 overexpression significantly reduced THCA cell proliferation. (c) qRT-PCR analysis of CATIP-AS1 expression after overexpressing THCA cells with CATIP-AS1. CATIP-AS1 expression was significantly upregulated. (d) Colony formation assay analysis of THCA cell proliferative ability after transfection with pcDNA3.1-CATIP-AS1. Overexpression of CATIP-AS1 reduced the number of colonies formed in THCA cell. (e) Apoptosis analysis of THCA cells after overexpression with CATIP-AS1. Overexpression of CATIP-AS1 increased THCA cell apoptotic rate.

To gain a better understanding of the regulatory role of CATIP-AS1 in THCA, an *in vivo* functional study was carried out using THCA cell lines. First, the relative expression of CATIP-AS1 in the THCA cell line BCPAP, IHH4, 8505C, BHT-101, and normal human thyroid cell line, Nthy-ori3-1, was established by qRT-PCR analysis. Then biological function of overexpressed CATIP-AS1 was examined in the THCA cell line through CCK-8 assay, colony formation assay, apoptosis test. Results showed that CATIP-AS1 was significantly downregulated in the THCA cell lines relative to the normal ones (*p* < 0.01, Figure 2(a)). Besides, the BCPAP and BHT-101 cell lines had the lowest CATIP-AS1 expression level and were chosen for subsequent molecular experiments. Our qRT-PCR analysis confirmed that CATIP-AS1 overexpression plasmid significantly upregulated the expression level of CATIP-AS1 in BCPAP and BHT-101 THCA cell line after transfection (*p* < 0.01, Figure 2(b)). Both CCK-8 and colony formation assay showed that the overexpression of CATIP-AS1 significantly inhibits THCA cell proliferation compared to the control (*p* < 0.01, Figure 2(c) and Figure 2(d)). Furthermore, we found that the apoptosis of THCA cell lines transfected with pcDNA3.1-CATIP-AS1 was markedly increased in comparison to the control vector group (*p* < 0.01, Figure 2(e)).

### CATIP-AS1 suppresses the migration of thyroid cancer cell lines via the EMT pathway

**Figure 3.**
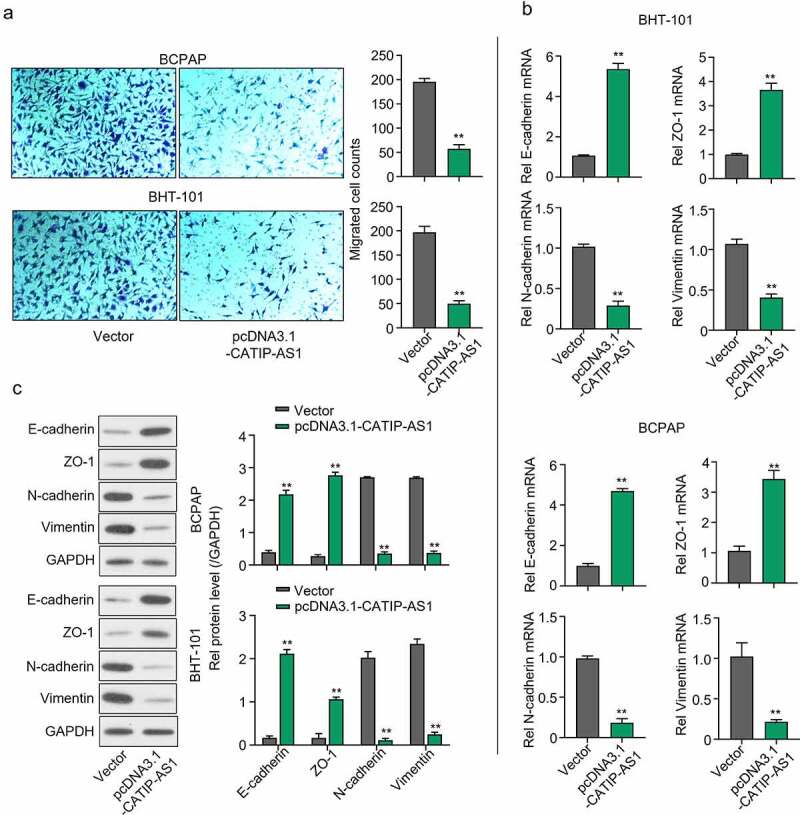
CATIP-AS1 suppresses the migration of THCA cell lines via the EMT pathway. (a) Transwell assay for the functional effect of CATIP-AS1 on the migration ability of the THCA cell lines. The migrative ability of THCA cell lines were inhibited after overexpression with CATIP-AS1. (b) Detection of EMT-related genes mRNA expression using qRT-PCR. E-cadherin and ZO-1 mRNA expression was markedly increased in both THCA cell lines overexpressed with CATIP-AS1 while the expression of N-cadherin and Vimentin was significantly repressed (c) Western blot analysis of EMT-related genes protein expression. E-cadherin and ZO-1 protein expression increased after overexpressing CATIP-AS1 in the THCA cell lines while N-cadherin and Vimentin protein expression was significantly repressed.

A transwell assay was carried out to examine the functional effect of CATIP-AS1 overexpression on the migration of the THCA cell lines, and the result showed that the migration ability of the BCPAP and BHT-101 cell lines were inhibited after transfection with pcDNA3.1-CATIP-AS1 (*p* < 0.01, Figure 3(a)). Furthermore, to determine the mRNA and protein expression of genes related to the EMT pathway, qRT-PCR and Western blot analysis were performed, respectively. We found that the mRNA and protein expression level of E-cadherin and ZO-1 markedly increased in both THCA cells overexpressed with CATIP-AS1 relative to the empty vector group, while the expression of N-cadherin, and Vimentin was significantly repressed (*p* < 0.01, Figure 3(b) and Figure 3(c)).

To further explore the in vivo function of CATIP-AS1, we used the BCPAP cells transfected with pcDNA3.1-CATIP-AS1 xenograft nude mouse model. Compared with the control group, CATIP-AS1 overexpression dramatically reduced the tumor growth (Figure S1A). Tumor weights were lighter at the endpoint in the presence of pcDNA3.1-CATIP-AS1 (Figure S1B). Above all, the overexpression of CATIP-AS1 inhibits THCA cell proliferation, EMT, as well as tumor growth and promotes cell apoptosis.

### CATIP-AS1 directly sponge miR-515-5p in thyroid cancer cells

**Figure 4.**
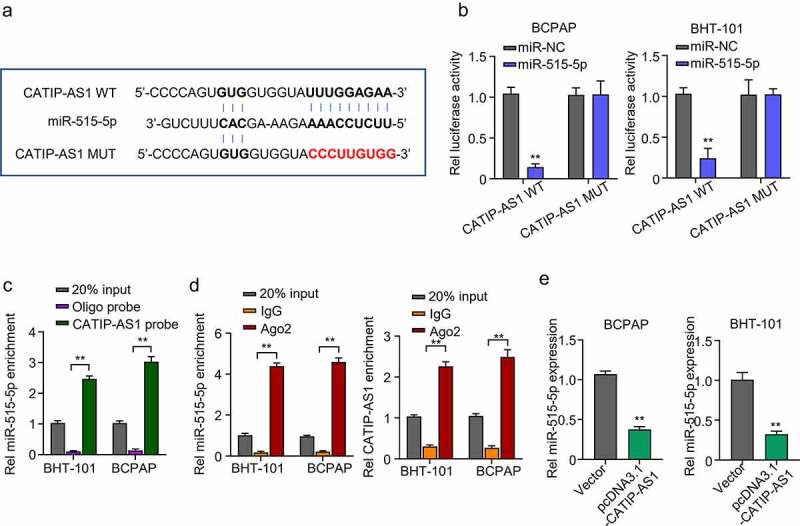
CATIP-AS1 directly sponge miR-515-5p in THCA cells (a) lncbase prediction result of CATIP-AS1 target miRNA. CATIP-AS1 sequence contained miR-515-5p seed region nucleotide sequences and could directly sponge miR-515-5p and inhibit its regulatory ability on its target mRNA in THCA cell. (b) Dual-Luciferase reporter assay to validate CATIP-AS1 and miR-515-5p binding ability. The miR-515-5p mimics significantly inhibited the luciferase activity of THCA cell lines co-transfected with CATIP-AS1 wild type (WT) plasmid but had no significant inhibiting effect on that of cells co-transfected with the CATIP-AS1 mutant type (Mut) plasmid. (c) Biotinylated RNA-pulldown assay. CATIP-AS1 probe significantly pull down more miR-515-5p in the THCA cell lines than the ordinary Oligo probe. (d) RIP-qRT-PCR analysis shows that more miR-515-5p and CATIP-AS1 was enriched in the Ago2 protein group compared to the IgG group. (e) QRT-PCR analysis of the relative expression of miR-515-5p in the THCA cell lines after CATIP-AS1 overexpression. The miR-515-5p expression was significantly inhibited.

After elucidating the possible role of CATIP-AS1 in THCA, we further sought to identify the plausible mechanism through which CATIP-AS1 performs its role in THCA. To achieve this, Lncbase was used for bioinformatics prediction of CATIP-AS1 target miRNA. The prediction results showed that CATIP-AS1 sequence contained nucleotide sequences found in the miR-515-5p seed region and could directly sponge miR-515-5p, inhibiting its regulatory ability on its target mRNA in THCA cell (Figure 4(a)). To confirm our result, we performed a dual-luciferase reporter assay. A biotinylated RNA pull-down assay was also carried out to examine CATIP-AS1 ability to pull down miR-515-5p in THCA cells, while RIP-qRT-PCR assay was used to examine CATIP-AS1-miR-515-5p binding ability. Luciferase assay revealed that miR-515-5p mimics significantly inhibited the luciferase activity of THCA cell lines (BCPAP and BHT-101) co-transfected with CATIP-AS1 wild type (WT) (CATIP-AS1-WT) plasmid relative to miR-NC while it had no significant inhibiting effect on the luciferase activity of those co-transfected with the CATIP-AS1 mutant type (Mut) (CATIP-AS1-MUT) plasmid (*p* < 0.01, Figure 4(b)). Further results showed that the biotinylated CATIP-AS1 probe could significantly pull down more hsa-miR-515-5p in BCPAP and BHT-101 cell lines than the ordinary Oligo probe (*p* < 0.01, Figure 4(c)). Similarly, through RIP-qRT-PCR analysis, we found that more miR-515-5p and CATIP-AS1 were enriched in the Ago2 protein group compared to the IgG group, further validating our bioinformatics prediction result (*p* < 0.01, Figure 4(d)). Moreover, the overexpression of CATIP-AS1 significantly inhibited miR-515-5p relative expression in the THCA cell lines (*p* < 0.01, Figure 4(e)).

### SMAD4 is a regulatory target of miR-515-5p in thyroid cancer cells

**Figure 5.**
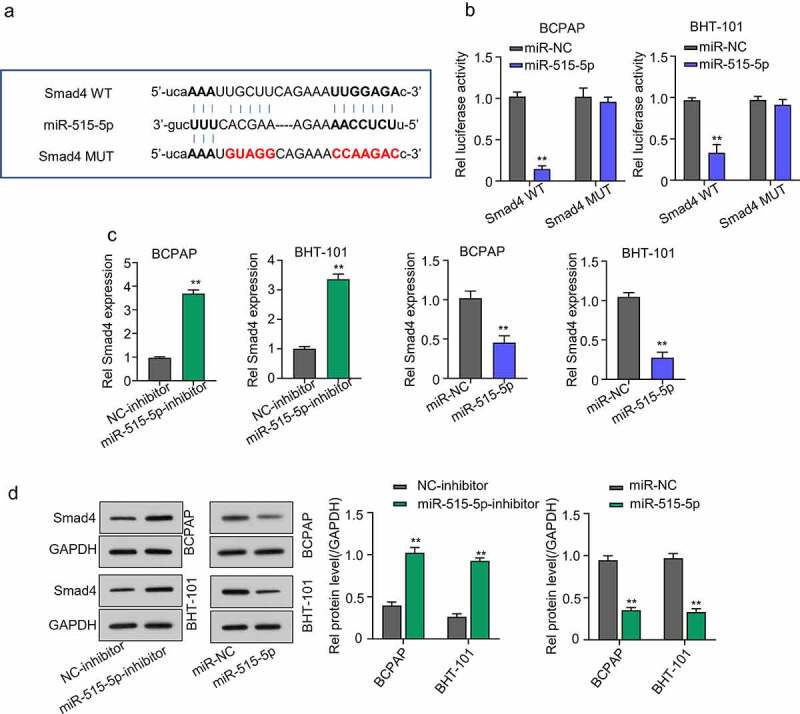
Smad4 is a regulatory target of miR-515-5p in THCA cells. (a) Starbase analysis predicted SMAD4 mRNA as a potential target miR-515-5p in THCA cells (b) Dual-Luciferase reporter assay. The luciferase activity of THCA cell lines co-transfected with miR-515-5p and SMAD4 wild type (WT) plasmid was significantly inhibited while that of cells co-transfected with SMAD4 mutant type (Mut) plasmid was not. (c) QRT-PCR analysis to measure the expression of SMAD4 mRNA after transfecting the THCA cell lines with miR-515-5p inhibitor or mimics. SMAD4 mRNA expression was significantly increased after miR-515-5p inhibition and was markedly repressed after overexpressing miR-515-5p (d) Western blot analysis. MiR-515-5p inhibition increased SMAD4 protein expression while miR-515-5p overexpression reduced it.

Through Starbase database, we were also able to predict SMAD4 as a potential miR-515-5p target mRNA in THCA cells (Figure 5(a)). Validation result showed that the luciferase activity of THCA cell lines co-transfected with miR-515-5p and SMAD4-WT plasmid was significantly repressed compared to those co-transfected with miR-NC and SMAD4-WT and CATIP-AS1-MUT-, confirming that miR-515-5p could bind to and regulate SMAD4 mRNA. Contrarily, no significant inhibition of luciferase activity was observed in THCA cell lines after mutating the binding site of miR-515-5p in SMAD4 and co-transfection (*p* < 0.01, Figure 5(b)). QRT-PCR and Western blot analysis further confirmed that miR-515-5p inhibition significantly increased the relative expression of SMAD4 mRNA and protein in both THCA cell lines, while the overexpression miR-515-5p markedly repressed the SMAD4 expression (*p* < 0.01, Figure 5(c) and Figure 5(d)).

### *CATIP-AS1 suppresses the migration of thyroid cancer cell* in vivo *via miR-515-5p/SMAD4 pathway*

**Figure 6.**
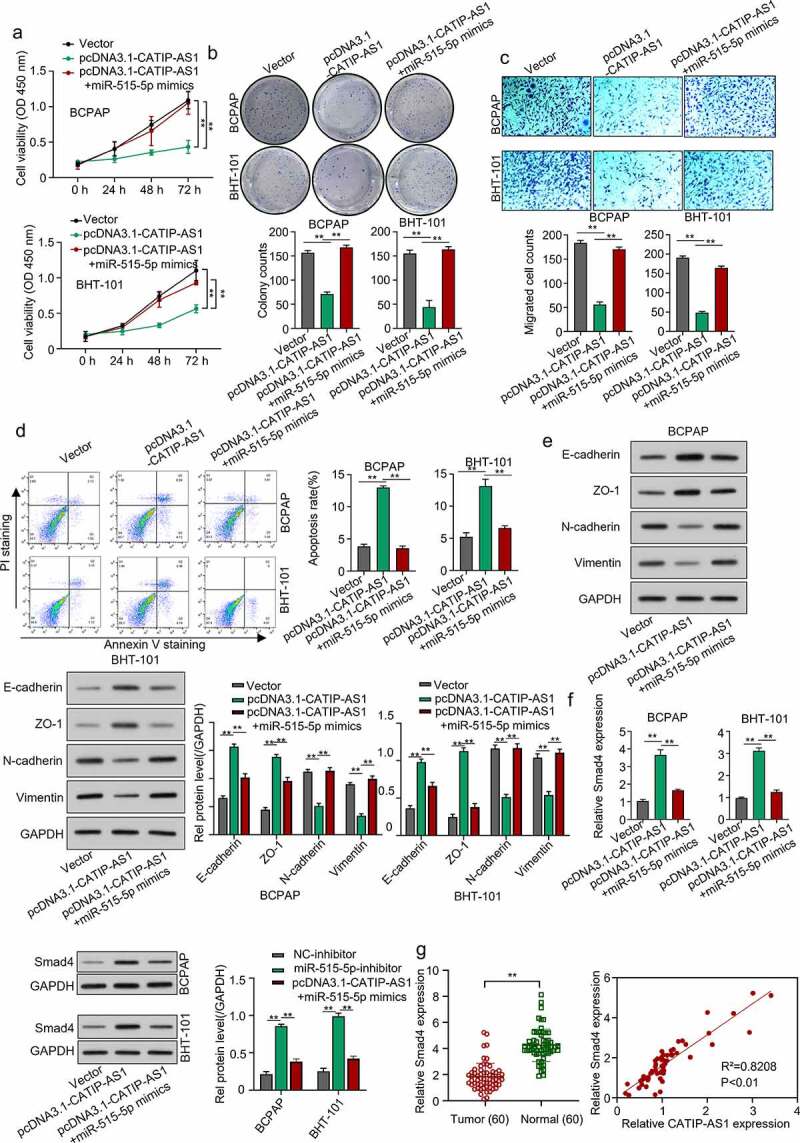
CATIP-AS1 suppresses the migration of THCA cell in vivo via miR-515-5p/SMAD4 pathway. (a) CCK-8 analysis of THCA cell proliferation after CATIP-AS1 and miR-515-5p overexpression. The cell proliferation was restored by miR-515-5p co-transfection (b) Colony formation assay. Co-transfecting miR-515-5p mimics reduced the number of colonies formed in the THCA cell lines. (c) Transwell assay. THCA cell migrative ability was restored by miR-515-5p mimics (d) Cell apoptosis assay. MiR-515-5p mimics reduced the apoptosis of THCA cell line (e) Western blot analysis of E-cadherin, ZO-1, N-cadherin, and Vimentin protein expression level in THCA cell lines after overexpressing CATIP-AS1 and co-transfection with miR-515-5p (f) SMAD4 expression level in THCA cell lines measured by qRT-PCR and Western blot analysis, after CATIP-AS1 and miR-515-5p overexpression. The relative expression of SMAD4 was significantly reduced after co-transfecting THCA cell lines with miR-515-5p mimics (g) QRT-PCR analysis of SMAD4 expression in THCA tumor samples and its association with CATIP-AS1 expression. SMAD4 was significantly upregulated in THCA samples compared to the normal ones and its expression was positively correlated with CATIP-AS1 expression.

To validate the regulatory mechanism of CATIP-AS1 in THCA, the BCPAP and BHT-101 cell lines were overexpressed with CATIP-AS1 and miR-515-5p. After which the proliferation, migration, and apoptosis rate of the cells were examined. The expression level of the EMT markers needed to be measured through Western blotting. Cell proliferation result showed that overexpressing CATIP-AS1 significantly reduced the proliferative ability of the THCA cell lines, which were restored after co-transfection with miR-515-5p mimics (*p* < 0.01, Figure 6(a) and Figure 6(b)). Migration ability of the THCA cell was also significantly inhibited after transfection with pcDNA3.1-CATIP-AS1 but reversed after co-transfection with miR-515-5p mimics (*p* < 0.01, Figure 6(c)). Similarly, we observed a significant increase in the apoptosis rate of THCA cell lines after transfecting with pcDNA3.1-CATIP-AS1. This was, however, reduced after co-transfection with miR-515-5p mimics (*p* < 0.01, Figure 6(d)). Furthermore, we found that E-cadherin and ZO-1 protein expression levels were markedly increased in the THCA cell lines overexpressed with CATIP-AS1 and significantly repressed by miR-515-5p mimics. While N-cadherin and Vimentin expression was repressed by overexpressed CATIP-AS1 and restored after co-transfection with miR-515-5p mimics, suggesting an EMT-induced regulation of THCA cells (Figure 6(e)). We also examined SMAD4 expression level in THCA cell lines after transfection and found that its relative expression was significantly increased in the cell after overexpressing CATIP-AS1. Subsequent co-transfection of the cell with miR-515-5p mimics, however, repressed SMAD4 mRNA and protein expression (*p* < 0.01, figure 6(f)). Interestingly, SMAD4 was found to be significantly downregulated in THCA samples relative to the normal ones and its expression was positively correlated with CATIP-AS1 expression (*p* < 0.01, Figure 6(g)).

## Discussion

THCA still remains a malignancy of extreme concern to human health. And even though there has been an increase in the 5-year survival rate, due to the early detection and treatment, which are essential for patients’ survival, there is still increasing cases across the globe [33]. In search of better noninvasive means of treating THCA, various research has focused on identifying putative genes or regulatory ncRNA that could serve as useful biomarkers for detecting THCA and a probable therapeutic drug target [34]. Despite this effort, the function of many putative genes or regulatory ncRNA remains unknown including their mechanism of regulation. Thus, the primary purpose of conducting this study was to determine the regulatory role of the CATIP-AS1 in THCA and its pathway of regulation. The results showed that CATIP-AS1 is significantly downregulated in THCA tissue data from GEO and clinical samples when compared to the normal healthy tissue samples or data and was associated with poor THCA patient’s prognosis. This result also coincides with a recent computational study by Rao *et al.* [20] which analyzed THCA tissue dataset from the GEO and GEPIA databases and reported that the CATIP-AS1 was the most significantly downregulated lncRNA in the analyzed data. However, the role of CATIP-AS1 was not elucidated in the study, neither was the mechanism of regulation well understood. Therefore, in our study, THCA cell lines were used only for various molecular and cellular biology experiments to better understand CATIP-AS1 role and mechanism of regulation in THCA. First, we evaluated the functional effect of overexpressed CATIP-AS1 in the THCA cell lines. Interestingly, we found that the overexpression of CATIP-AS1 could significantly reduce the THCA cell viability, and colony formation, but increased the cell apoptosis, and cell growth, suggesting a potential therapeutic property of the CATIP-AS1 in THCA.

The lncRNAs have been studied over the years and reported being involved in chromatin modification or regulation, protein, and RNA interaction, and play a key role in the proliferation, migration, apoptosis, EMT, and metastasis of cancer cells [35]. Specifically, a recent study on papillary THCA reported that the overexpression of the lncRNA TUG1 promotes papillary thyroid tumor cell proliferation, migration, and EMT formation by regulating the miR-145/ZEB1 axis [35]. Another study also showed that the overexpression of TUSC7 can significantly inhibit the proliferation, migration, invasion, and EMT of colorectal cancer cell [36]. Similarly, Yang *et al.* demonstrated that the lncRNA GHET1 is overexpressed in osteosarcoma and silencing GHET1 significantly inhibits the proliferation, migration, invasion, and EMT of osteosarcoma cells [37]. There are other studies that regulate thyroid cancer with similar signaling pathways, such as lncRNA DUXAP8 promotes the cell proliferation, migration, and invasion of papillary thyroid carcinoma via miR-223-3p mediated regulation of CXCR4 [38]. LncRNA AFAP-AS1 promotes anaplastic thyroid cancer progression by sponging miR-155-5p through ETS1/ERK pathway [22]. However, the biological function of CATIP-AS1 in various cancers has not been well explored. Furthermore, various lncRNAs have also been implicated in the dysregulation of EMT pathway in cancer cells, which consequently promote the development and progression of different cancerous cells in humans. Accumulating evidence shows that EMT-like events play a major role in the progression of various tumors and transformation of diverse malignant cells, especially when the cancerous cells take on the invasive and metastatic ability of mesenchymal cell [39]. One major molecular mechanism responsible for such event is the repression of cell adhesion molecules like the E-cadherin and ZO-1 and the upregulation of N-cadherin and Vimentin in the EMT pathway [40]. Notably, the modulation of this pathway could be successful in treating THCA [41]. For instance, the upregulation of lncRNA AFAP1-AS1 in osteosarcoma (OS) has been showed to promote the tumorigenesis, EMT, and consequent progression of OS cells through the regulation of the RhoC/ROCK1/p38MAPK/Twist1 axis [42]. It has further been shown to promote the EMT of triple-negative breast cancer cells and their development by regulating the Wnt/β-Catenin signaling pathway [43]. Besides, the dysregulated expression of EMT-related proteins in triptolide-induced polyploid giant cancer cells and their daughter cells reduced the migration, invasive, and proliferative capacity of the cells [44]. Therefore, in this study, the aberrant expression of epithelial and mesenchymal markers was examined. Our result indicated that the overexpression of CATIP-AS1 significantly increased the expression of E-cadherin and ZO-1 but decreased N-cadherin and Vimentin expression, indicating that the overexpression of CATIP-AS1 could partly regulate the EMT-induced protein and the resulting tumorigenesis and progression of THCA. Using the lncbase and starbase, we predicted that the CATIP-AS1 could inhibit the regulatory effect of miR-515-5p on SMAD4, and this pathway might be responsible for the regulation of the EMT-induced protein in THCA cell and the resulting proliferation, migration, and metastasis.

Our results further revealed that the overexpression of CATIP-AS1 inhibited the miR-515-5p expression and upregulated the mRNA and protein expression level of SMAD4. Moreover, the overexpression of CATIP-AS1 was also found to regulate the mRNA and protein expression level of the EMT-related gene, consequently reducing the proliferation, and migration of the THCA cell lines. Altogether, this data suggests that the overexpressed-CATIP-AS1-induced upregulation of SMAD4 might be responsible for the upregulation of E-cadherin and ZO-1 and the downregulation of N-cadherin and Vimentin in the EMT pathway. The miR-515-5p is a novel miRNA whose function has not meant elucidated. It has been reported to be downregulated in keloid [45] and also in proliferating colorectal cancer cell lines incubated with *Fusobacterium nucleatum* [46]. Increasing evidence shows that the loss or inactivation of the SMAD4 gene, a critical mediator of the transforming growth factor beta (TGF-β) signaling pathways, indicates a poor prognosis in various cancers, especially pancreatic cancer [47]. Summarily, this study demonstrated that the dysregulation of CATIP-AS1 in THCA cells regulated the proliferation, migration, apoptosis, and growth of THCA cells through the EMT pathway.

## Conclusion

In this study, we found that CATIP-AS1 is significantly downregulated in THCA. We experimentally verified whether the CATIP-AS1 can target miR-515-5p in THCA cells to regulate the expression of SMAD4 and ultimately the EMT-pathway. We found that the overexpression of CATIP-AS1 could play a role in inhibiting the occurrence and development of THCA. CATIP-AS1 might be a novel therapeutic target for treating THCA.

## Supplementary Material

Supplemental Material

## Data Availability

All supporting data of this work, which are not available to public because of ethical restrictions, are available from the corresponding author upon request.
