# Supplementary figures and images for: Long non-coding RNA CATIP antisense RNA 1 (lncRNA CATIP-AS1) downregulation contributes to the progression and metastasis of thyroid cancer via epithelial–mesenchymal transition (EMT) pathway
Source: Bioengineered. 2022 Mar 10;13(3):7592–606. doi: 10.1080/21655979.2022.2047400 (PMC8973971; doi:10.1080/21655979.2022.2047400)

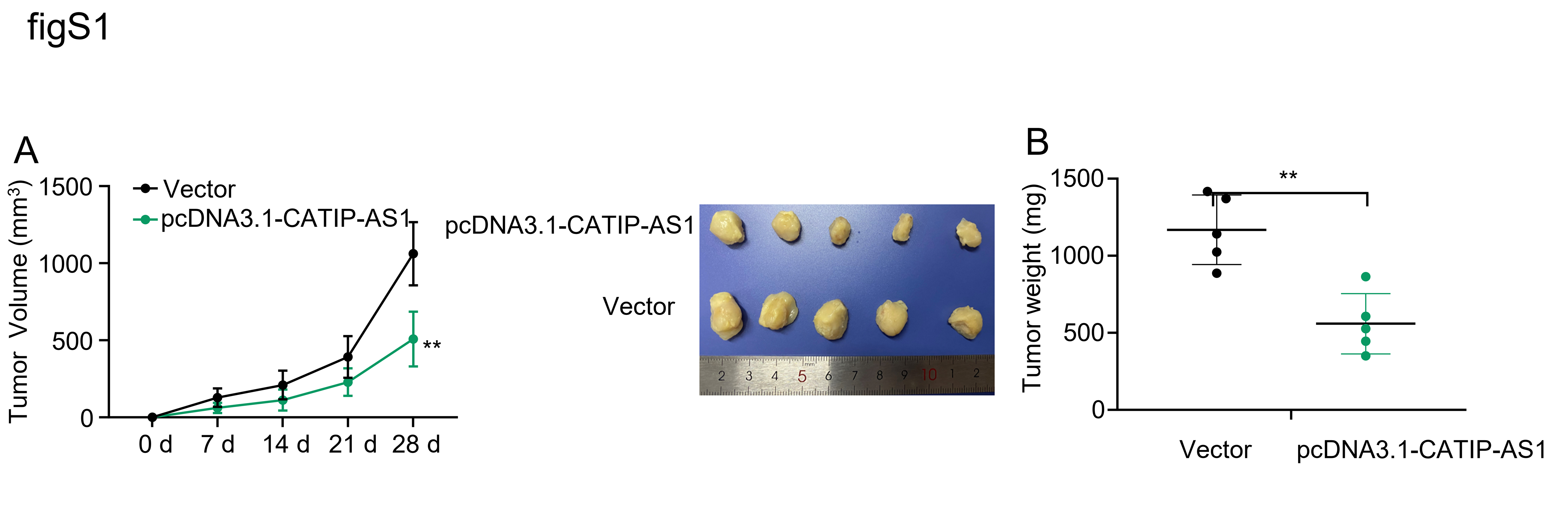

Supplement: Supplemental Material [file KBIE_A_2047400_SM2038.tif]
